# Effects of Polyphenol-Rich Foods on Human Health

**DOI:** 10.3390/nu10081089

**Published:** 2018-08-14

**Authors:** Giuseppe Grosso

**Affiliations:** NNEdPro Global Centre for Nutrition and Health, St John’s Innovation Centre, Cambridge CB4 0WS, UK; giuseppe.grosso@studium.unict.it; Tel.: +39-095-378-2182

**Keywords:** polyphenol-rich foods, polyphenol, flavonoids, human health, evidence-based

## Abstract

Recent evidence has suggested that polyphenol-rich foods intake may be associated with decreased risk of chronic diseases. The Special Issue “Effects of Polyphenol-Rich Foods on Human Health” comprised 64 peer-reviewed papers on the most recent evidence regarding the dietary intake of polyphenols and polyphenol-rich foods, as well as their effect toward the prevention and treatment of non-communicable diseases. Original contributions and literature reviews demonstrated the potential protective effects of polyphenol-rich foods and their extracts toward cardiovascular diseases, certain cancers, and neurodegenerative diseases, mostly through anti-oxidant and chemo-preventive properties.

Over the last years, polyphenol-rich foods and polyphenols have received great attention due to their potential beneficial effects toward human health. Contained not only in fruits and vegetables, but also in whole-grains, nuts, olive oil and beverages such as coffee and tea, they are characteristic components of healthy dietary patterns [1]. Recent evidence has proposed that a higher dietary intake of polyphenols may be inversely associated with overall and CVD-related mortality [2], certain cancers [3], cardiovascular diseases (CVDs) [4], anthropometric measures [5], and mood disorders [6,7]. Thus, several molecular mechanisms have been suggested, however, one of the most investigated biological activities of polyphenols is their antioxidant activity.

This Special Issue “Effects of Polyphenol-Rich Foods on Human Health” comprises 64 peer-reviewed papers, including 43 original research papers [8,9,10,11,12,13,14,15,16,17,18,19,20,21,22,23,24,25,26,27,28,29,30,31,32,33,34,35,36,37,38,39,40,41,42,43,44,45,46,47,48,49,50], one case-report [51] and 20 literature reviews [52,53,54,55,56,57,58,59,60,61,62,63,64,65,66,67,68,69,70,71]. They report on the most recent evidence regarding the dietary intake of polyphenols and polyphenol-rich foods, as well as their effect toward the prevention and treatment of non-communicable diseases.

The Special Issue included systematic reviews and meta-analysis summarizing the level of evidence on both polyphenols and polyphenol-rich foods. In particular, published articles demonstrated that a moderate intake of coffee is inversely associated with the risk of hypertension [23], and cancer, including liver [21], endometrial [58] and postmenopausal breast cancer [28]. Marventano et al. reported that a high dietary intake of whole-grain foods is able to improve postprandial glucose and insulin homeostasis in healthy individuals [61]. Moreover, the results of a comprehensive meta-analysis on randomized clinical trials showed that intake of flavanol-containing products is associated with body composition and blood lipids level [56]. Shivappa et al. reported that a diet with lower inflammatory potential in inversely associated with risk of CVD and colorectal cancer [65,66]. Finally, a meta-analysis exploring the association between polyphenol biomarkers and CVD and mortality, revealed that higher enterolactone concentrations were associated with all-cause and CVD mortality risk [63].

Epidemiological and clinical studies reported the following associations of polyphenols and polyphenol food extracts: silibinin and non-alcoholic fatty liver disease [9,39], resveratrol and hepatic encephalopathy [33], coffee polyphenols and cardiovascular risk factors [34], flavonoids and ventilator function [20], tea and stress and sleep quality (depending on caffeine content) [46], tea and glycemic response [44], polyphenols and obesity [24], phenolic acids and hypertension [22], healthy diet and neurofibromatosis [51].

Importantly, numerous reviews summarized the evidence regarding polyphenols and polyphenol-rich foods and their association with NCDs related to oxidative stress and inflammation, including CVDs, diabetes, hypertension, obesity, certain cancers and neurodegenerative diseases [19,45,52,53,54,55,57,59,60,62,64,67,68,69,70,71].

Several contributions investigated polyphenol metabolism and transformation into bioavailable molecules and underlined the importance of the clarification of the metabolic fate of polyphenols and their bioavailability in order to fully understand the molecular forms responsible for the preventive actions of polyphenols [13,15,17,26,35,36,43].

Polyphenols have been demonstrated to be responsible for the anti-inflammatory and antioxidant properties of potentially functional foods. Numerous studies explored in detail the molecular mechanisms underlying the protective effects of polyphenols, including antioxidant capacity, that can be attributed to the regulation of redox enzymes through reducing reactive oxygen species (ROS) production and modulation of the II-phase enzymes responsible for the cellular oxidative response; other studies explored the chemo-preventive effects of polyphenols, including the elimination of carcinogenic agents, the modulation of pathways responsible for cancer cell signaling and cell cycle progression, and in the promotion of apoptosis [8,10,11,12,14,16,18,25,27,29,30,31,32,37,38,40,41,42,47,48,49,50].

As a guest editor, I would like to acknowledge all the authors for their valuable contributions and the reviewers for their constructive remarks. Special thanks to the publishing team of the journal *Nutrients* for their professional help in the completion of this Special Issue.

## References

[1] Grosso G., Bella F., Godos J., Sciacca S., Del Rio D., Ray S., Galvano F., Giovannucci E.L. (2017). Possible role of diet in cancer: Systematic review and multiple meta-analyses of dietary patterns, lifestyle factors, and cancer risk. Nutr. Rev..

[2] Grosso G., Micek A., Godos J., Pajak A., Sciacca S., Galvano F., Giovannucci E.L. (2017). Dietary flavonoid and lignan intake and mortality in prospective cohort studies: Systematic review and dose-response meta-analysis. Am. J. Epidemiol..

[3] Grosso G., Godos J., Lamuela-Raventos R., Ray S., Micek A., Pajak A., Sciacca S., D’Orazio N., Del Rio D., Galvano F. (2017). A comprehensive meta-analysis on dietary flavonoid and lignan intake and cancer risk: Level of evidence and limitations. Mol. Nutr. Food Res..

[4] Wang X., Ouyang Y.Y., Liu J., Zhao G. (2014). Flavonoid intake and risk of cvd: A systematic review and meta-analysis of prospective cohort studies. Br. J. Nutr..

[5] Carnauba R.A., Chaves D.F., Baptistella A.B., Paschoal V., Naves A., Buehler A.M. (2017). Association between high consumption of phytochemical-rich foods and anthropometric measures: A systematic review. Int. J. Food Sci. Nutr..

[6] Chang S.C., Cassidy A., Willett W.C., Rimm E.B., O’Reilly E.J., Okereke O.I. (2016). Dietary flavonoid intake and risk of incident depression in midlife and older women. Am. J. Clin. Nutr..

[7] Godos J., Castellano S., Ray S., Grosso G., Galvano F. (2018). Dietary polyphenol intake and depression: Results from the mediterranean healthy eating, lifestyle and aging (meal) study. Molecules.

[8] Al-Dosari D.I., Ahmed M.M., Al-Rejaie S.S., Alhomida A.S., Ola M.S. (2017). Flavonoid naringenin attenuates oxidative stress, apoptosis and improves neurotrophic effects in the diabetic rat retina. Nutrients.

[9] Amato A., Caldara G.F., Nuzzo D., Baldassano S., Picone P., Rizzo M., Mule F., Di Carlo M. (2017). Nafld and atherosclerosis are prevented by a natural dietary supplement containing curcumin, silymarin, guggul, chlorogenic acid and inulin in mice fed a high-fat diet. Nutrients.

[10] Bijak M., Dziedzic A., Synowiec E., Sliwinski T., Saluk-Bijak J. (2017). Flavonolignans inhibit il1-beta-induced cross-talk between blood platelets and leukocytes. Nutrients.

[11] Bijak M., Synowiec E., Sitarek P., Sliwinski T., Saluk-Bijak J. (2017). Evaluation of the cytotoxicity and genotoxicity of flavonolignans in different cellular models. Nutrients.

[12] Borowska S., Brzoska M.M., Galazyn-Sidorczuk M., Rogalska J. (2017). Effect of an extract from aronia melanocarpa l. Berries on the body status of zinc and copper under chronic exposure to cadmium: An in vivo experimental study. Nutrients.

[13] Bresciani L., Martini D., Mena P., Tassotti M., Calani L., Brigati G., Brighenti F., Holasek S., Malliga D.E., Lamprecht M. (2017). Absorption profile of (poly)phenolic compounds after consumption of three food supplements containing 36 different fruits, vegetables, and berries. Nutrients.

[14] Camps-Bossacoma M., Franch A., Perez-Cano F.J., Castell M. (2017). Influence of hesperidin on the systemic and intestinal rat immune response. Nutrients.

[15] Chavez-Suarez K.M., Ortega-Velez M.I., Valenzuela-Quintanar A.I., Galvan-Portillo M., Lopez-Carrillo L., Esparza-Romero J., Saucedo-Tamayo M.S., Robles-Burgueno M.R., Palma-Duran S.A., Gutierrez-Coronado M.L. (2017). Phytoestrogen concentrations in human urine as biomarkers for dietary phytoestrogen intake in mexican women. Nutrients.

[16] Choi J., Kim K.J., Koh E.J., Lee B.Y. (2017). Gelidium elegans regulates the ampk-prdm16-ucp-1 pathway and has a synergistic effect with orlistat on obesity-associated features in mice fed a high-fat diet. Nutrients.

[17] Fang Y., Cao W., Xia M., Pan S., Xu X. (2017). Study of structure and permeability relationship of flavonoids in caco-2 cells. Nutrients.

[18] Figueiredo E.A., Alves N.F.B., Monteiro M.M.O., Cavalcanti C.O., Silva T., Silva T., Braga V.A., Oliveira E.J. (2017). Antioxidant and antihypertensive effects of a chemically defined fraction of syrah red wine on spontaneously hypertensive rats. Nutrients.

[19] Ganesan K., Xu B. (2017). A critical review on polyphenols and health benefits of black soybeans. Nutrients.

[20] Garcia-Larsen V., Thawer N., Charles D., Cassidy A., van Zele T., Thilsing T., Ahlstrom M., Haahtela T., Keil T., Matricardi P.M. (2018). Dietary intake of flavonoids and ventilatory function in european adults: A ga(2)len study. Nutrients.

[21] Godos J., Micek A., Marranzano M., Salomone F., Rio D.D., Ray S. (2017). Coffee consumption and risk of biliary tract cancers and liver cancer: A dose-response meta-analysis of prospective cohort studies. Nutrients.

[22] Godos J., Sinatra D., Blanco I., Mule S., La Verde M., Marranzano M. (2017). Association between dietary phenolic acids and hypertension in a mediterranean cohort. Nutrients.

[23] Grosso G., Micek A., Godos J., Pajak A., Sciacca S., Bes-Rastrollo M., Galvano F., Martinez-Gonzalez M.A. (2017). Long-term coffee consumption is associated with decreased incidence of new-onset hypertension: A dose-response meta-analysis. Nutrients.

[24] Guo X., Tresserra-Rimbau A., Estruch R., Martinez-Gonzalez M.A., Medina-Remon A., Fito M., Corella D., Salas-Salvado J., Portillo M.P., Moreno J.J. (2017). Polyphenol levels are inversely correlated with body weight and obesity in an elderly population after 5 years of follow up (the randomised predimed study). Nutrients.

[25] Kim S.N., Kwon H.J., Akindehin S., Jeong H.W., Lee Y.H. (2017). Effects of epigallocatechin-3-gallate on autophagic lipolysis in adipocytes. Nutrients.

[26] Kubow S., Iskandar M.M., Melgar-Bermudez E., Sleno L., Sabally K., Azadi B., How E., Prakash S., Burgos G., Felde T.Z. (2017). Effects of simulated human gastrointestinal digestion of two purple-fleshed potato cultivars on anthocyanin composition and cytotoxicity in colonic cancer and non-tumorigenic cells. Nutrients.

[27] Kwon E.Y., Lee J., Kim Y.J., Do A., Choi J.Y., Cho S.J., Jung U.J., Lee M.K., Park Y.B., Choi M.S. (2017). Seabuckthorn leaves extract and flavonoid glycosides extract from seabuckthorn leaves ameliorates adiposity, hepatic steatosis, insulin resistance, and inflammation in diet-induced obesity. Nutrients.

[28] Lafranconi A., Micek A., De Paoli P., Bimonte S., Rossi P., Quagliariello V., Berretta M. (2018). Coffee intake decreases risk of postmenopausal breast cancer: A dose-response meta-analysis on prospective cohort studies. Nutrients.

[29] Li W.F., Yang K., Zhu P., Zhao H.Q., Song Y.H., Liu K.C., Huang W.F. (2017). Genistein ameliorates ischemia/reperfusion-induced renal injury in a sirt1-dependent manner. Nutrients.

[30] Liu C., Ma J., Sun J., Cheng C., Feng Z., Jiang H., Yang W. (2017). Flavonoid-rich extract of paulownia fortunei flowers attenuates diet-induced hyperlipidemia, hepatic steatosis and insulin resistance in obesity mice by ampk pathway. Nutrients.

[31] Liu W.Y., Liou S.S., Hong T.Y., Liu I.M. (2017). Protective effects of hesperidin (citrus flavonone) on high glucose induced oxidative stress and apoptosis in a cellular model for diabetic retinopathy. Nutrients.

[32] Ma Q., Guo Y., Sun L., Zhuang Y. (2017). Anti-diabetic effects of phenolic extract from rambutan peels (*Nephelium lappaceum*) in high-fat diet and streptozotocin-induced diabetic mice. Nutrients.

[33] Malaguarnera G., Pennisi M., Bertino G., Motta M., Borzi A.M., Vicari E., Bella R., Drago F., Malaguarnera M. (2018). Resveratrol in patients with minimal hepatic encephalopathy. Nutrients.

[34] Miranda A.M., Steluti J., Fisberg R.M., Marchioni D.M. (2017). Association between coffee consumption and its polyphenols with cardiovascular risk factors: A population-based study. Nutrients.

[35] Noh H., Freisling H., Assi N., Zamora-Ros R., Achaintre D., Affret A., Mancini F., Boutron-Ruault M.C., Flogel A., Boeing H. (2017). Identification of urinary polyphenol metabolite patterns associated with polyphenol-rich food intake in adults from four european countries. Nutrients.

[36] Quiros-Sauceda A.E., Chen C.O., Blumberg J.B., Astiazaran-Garcia H., Wall-Medrano A., Gonzalez-Aguilar G.A. (2017). Processing ‘ataulfo’ mango into juice preserves the bioavailability and antioxidant capacity of its phenolic compounds. Nutrients.

[37] Riccio G., Maisto M., Bottone S., Badolati N., Rossi G.B., Tenore G.C., Stornaiuolo M., Novellino E. (2017). Wnt inhibitory activity of malus pumila miller cv annurca and malus domestica cv limoncella apple extracts on human colon-rectal cells carrying familial adenomatous polyposis mutations. Nutrients.

[38] Rzepecka-Stojko A., Stojko J., Jasik K., Buszman E. (2017). Anti-atherogenic activity of polyphenol-rich extract from bee pollen. Nutrients.

[39] Salomone F., Barbagallo I., Godos J., Lembo V., Currenti W., Cina D., Avola R., D’Orazio N., Morisco F., Galvano F. (2017). Silibinin restores nad(+) levels and induces the sirt1/ampk pathway in non-alcoholic fatty liver. Nutrients.

[40] Schell J., Scofield R.H., Barrett J.R., Kurien B.T., Betts N., Lyons T.J., Zhao Y.D., Basu A. (2017). Strawberries improve pain and inflammation in obese adults with radiographic evidence of knee osteoarthritis. Nutrients.

[41] Selby-Pham S.N.B., Cottrell J.J., Dunshea F.R., Ng K., Bennett L.E., Howell K.S. (2017). Dietary phytochemicals promote health by enhancing antioxidant defence in a pig model. Nutrients.

[42] Sonoki H., Tanimae A., Endo S., Matsunaga T., Furuta T., Ichihara K., Ikari A. (2017). Kaempherol and luteolin decrease claudin-2 expression mediated by inhibition of stat3 in lung adenocarcinoma a549 cells. Nutrients.

[43] Spigoni V., Mena P., Fantuzzi F., Tassotti M., Brighenti F., Bonadonna R.C., Del Rio D., Dei Cas A. (2017). Bioavailability of bergamot (*Citrus bergamia*) flavanones and biological activity of their circulating metabolites in human pro-angiogenic cells. Nutrients.

[44] Suraphad P., Suklaew P.O., Ngamukote S., Adisakwattana S., Makynen K. (2017). The effect of isomaltulose together with green tea on glycemic response and antioxidant capacity: A single-blind, crossover study in healthy subjects. Nutrients.

[45] Szwajgier D., Borowiec K., Pustelniak K. (2017). The neuroprotective effects of phenolic acids: Molecular mechanism of action. Nutrients.

[46] Unno K., Noda S., Kawasaki Y., Yamada H., Morita A., Iguchi K., Nakamura Y. (2017). Reduced stress and improved sleep quality caused by green tea are associated with a reduced caffeine content. Nutrients.

[47] Venturi F., Sanmartin C., Taglieri I., Nari A., Andrich G., Terzuoli E., Donnini S., Nicolella C., Zinnai A. (2017). Development of phenol-enriched olive oil with phenolic compounds extracted from wastewater produced by physical refining. Nutrients.

[48] Yeh Y.T., Chiang A.N., Hsieh S.C. (2017). Chinese olive (*Canarium album* L.) fruit extract attenuates metabolic dysfunction in diabetic rats. Nutrients.

[49] Youn K., Yu Y., Lee J., Jeong W.S., Ho C.T., Jun M. (2017). Polymethoxyflavones: Novel beta-secretase (bace1) inhibitors from citrus peels. Nutrients.

[50] Yuan E., Duan X., Xiang L., Ren J., Lai X., Li Q., Sun L., Sun S. (2018). Aged oolong tea reduces high-fat diet-induced fat accumulation and dyslipidemia by regulating the ampk/acc signaling pathway. Nutrients.

[51] Esposito T., Schettino C., Polverino P., Allocca S., Adelfi L., D’Amico A., Capaldo G., Varriale B., Di Salle A., Peluso G. (2017). Synergistic interplay between curcumin and polyphenol-rich foods in the mediterranean diet: Therapeutic prospects for neurofibromatosis 1 patients. Nutrients.

[52] Amawi H., Ashby C.R., Samuel T., Peraman R., Tiwari A.K. (2017). Polyphenolic nutrients in cancer chemoprevention and metastasis: Role of the epithelial-to-mesenchymal (emt) pathway. Nutrients.

[53] Baiao D.D.S., de Freitas C.S., Gomes L.P., da Silva D., Correa A., Pereira P.R., Aguila E.M.D., Paschoalin V.M.F. (2017). Polyphenols from root, tubercles and grains cropped in brazil: Chemical and nutritional characterization and their effects on human health and diseases. Nutrients.

[54] Chin K.Y., Pang K.L. (2017). Therapeutic effects of olive and its derivatives on osteoarthritis: From bench to bedside. Nutrients.

[55] Danesi F., Ferguson L.R. (2017). Could pomegranate juice help in the control of inflammatory diseases?. Nutrients.

[56] González-Sarrías A., Combet E., Pinto P., Mena P., Dall’Asta M., Garcia-Aloy M., Rodríguez-Mateos A., Gibney E.R., Dumont J., Massaro M. (2017). A systematic review and meta-analysis of the effects of flavanol-containing tea, cocoa and apple products on body composition and blood lipids: Exploring the factors responsible for variability in their efficacy. Nutrients.

[57] Ho Y., Lin Y.S., Liu H.L., Shih Y.J., Lin S.Y., Shih A., Chin Y.T., Chen Y.R., Lin H.Y., Davis P.J. (2017). Biological mechanisms by which antiproliferative actions of resveratrol are minimized. Nutrients.

[58] Lafranconi A., Micek A., Galvano F., Rossetti S., Del Pup L., Berretta M., Facchini G. (2017). Coffee decreases the risk of endometrial cancer: A dose-response meta-analysis of prospective cohort studies. Nutrients.

[59] Lee Y.M., Yoon Y., Yoon H., Park H.M., Song S., Yeum K.J. (2017). Dietary anthocyanins against obesity and inflammation. Nutrients.

[60] Li Y., Li S., Meng X., Gan R.Y., Zhang J.J., Li H.B. (2017). Dietary natural products for prevention and treatment of breast cancer. Nutrients.

[61] Marventano S., Vetrani C., Vitale M., Godos J., Riccardi G., Grosso G. (2017). Whole grain intake and glycaemic control in healthy subjects: A systematic review and meta-analysis of randomized controlled trials. Nutrients.

[62] Mattera R., Benvenuto M., Giganti M.G., Tresoldi I., Pluchinotta F.R., Bergante S., Tettamanti G., Masuelli L., Manzari V., Modesti A. (2017). Effects of polyphenols on oxidative stress-mediated injury in cardiomyocytes. Nutrients.

[63] Rienks J., Barbaresko J., Nothlings U. (2017). Association of polyphenol biomarkers with cardiovascular disease and mortality risk: A systematic review and meta-analysis of observational studies. Nutrients.

[64] Sequeira I.R., Poppitt S.D. (2017). Unfolding novel mechanisms of polyphenol flavonoids for better glycaemic control: Targeting pancreatic islet amyloid polypeptide (IAPP). Nutrients.

[65] Shivappa N., Godos J., Hebert J.R., Wirth M.D., Piuri G., Speciani A.F., Grosso G. (2017). Dietary inflammatory index and colorectal cancer risk-a meta-analysis. Nutrients.

[66] Shivappa N., Godos J., Hebert J.R., Wirth M.D., Piuri G., Speciani A.F., Grosso G. (2018). Dietary inflammatory index and cardiovascular risk and mortality-a meta-analysis. Nutrients.

[67] Souza P.A.L., Marcadenti A., Portal V.L. (2017). Effects of olive oil phenolic compounds on inflammation in the prevention and treatment of coronary artery disease. Nutrients.

[68] Tang G.Y., Meng X., Li Y., Zhao C.N., Liu Q., Li H.B. (2017). Effects of vegetables on cardiovascular diseases and related mechanisms. Nutrients.

[69] Testai L., Calderone V. (2017). Nutraceutical value of citrus flavanones and their implications in cardiovascular disease. Nutrients.

[70] Zhang H., Ma Z.F. (2018). Phytochemical and pharmacological properties of capparis spinosa as a medicinal plant. Nutrients.

[71] Zhao C.N., Meng X., Li Y., Li S., Liu Q., Tang G.Y., Li H.B. (2017). Fruits for prevention and treatment of cardiovascular diseases. Nutrients.

